# Cryptic Polyketide Synthase Genes in Non-Pathogenic *Clostridium* SPP

**DOI:** 10.1371/journal.pone.0029609

**Published:** 2012-01-03

**Authors:** Swantje Behnken, Christian Hertweck

**Affiliations:** 1 Leibniz Institute for Natural Product Research and Infection Biology, Hans Knöll Institute, Jena, Germany; 2 Chair of Natural Product Chemistry, Friedrich Schiller University, Jena, Germany; The University of Hong Kong, China

## Abstract

Modular type I polyketide synthases (PKS) produce a vast array of bacterial metabolites with highly diverse biological functions. Notably, all known polyketides were isolated from aerobic bacteria, and yet no example has been reported for strict anaerobes. In this study we explored the diversity and distribution of PKS genes in the genus *Clostridium*. In addition to comparative genomic analyses combined with predictions of modular type I polyketide synthase (PKS) gene clusters in sequenced genomes of *Clostridium* spp., a representative selection of other species inhabiting a variety of ecological niches was investigated by PCR screening for PKS genes. Our data reveal that all studied pathogenic *Clostridium* spp. are devoid of putative PKS genes. In stark contrast, cryptic PKS genes are widespread in genomes of non-pathogenic *Clostridium* species. According to phylogenetic analyses, the *Clostridium* PKS genes have unusual and diverse origins. However, reverse transcription quantitative PCR demonstrates that these genes are silent under standard cultivation conditions, explaining why the related metabolites have been overlooked until now. This study presents clostridia as a putative source for novel bioactive polyketides.

## Introduction

Bacterial modular type I polyketide synthases (PKS) are giant multifunctional enzymes that are responsible for the biosynthesis of complex polyketides, such as macrolides, polyenes, and polyethers. These secondary metabolites possess a wide range of pharmaceutical properties including antibiotic, antifungal, antitumor, and immunosuppressive activities [1]. Until recently, only PKSs of a few bacterial genera, predominantly actinomycetes and myxobacteria, were the focus of investigation. However, the number of genera known to produce polyketides has recently expanded to include *Bacillus*, *Pseudomonas* and *Burkholderia* spp., for example [2], [3]. Genetic studies have yielded detailed information on the organization and function of the PKS genes involved in the biosynthesis of polyketides [1]. Bacterial modular PKS have been found to have significant homology to each other. Structurally PKS are encoded by a variable number of modules that perform a stepwise biosynthesis of carbon skeletons from simple activated acyl and malonyl acid units. Ketoacyl synthase (KS) domains in PKS are required for the condensation of extender units on a growing polyketide chain [2], [4]. The highly conserved nature of these KS domains can be employed to screen underexplored or neglected bacterial genome sequences for PKS genes to investigate the actual metabolic potential of hitherto neglected microorganisms, such as clostridia [3], [5].

Recently, we have discovered the first antibiotic, closthioamide, from the strictly anaerobic bacterium *Clostridium cellulolyticum* [6]. This result encouraged us to explore the virtually unexplored secondary metabolome of obligate anaerobes, specifically *Clostridium* spp., and screen their genome sequences for PKS genes.

Members of the genus *Clostridium* are gram-positive, obligately anaerobic bacteria and include prominent human pathogens, like *Clostridium botulinum*, and species extensively used for biofuel production, cellulose degradation, and other biotechnological applications [7]. Due to their relevance as pathogens or producer strains in biotechnology, 100 *Clostridium* genomes have already been sequenced and published (42 completed and 58 whole genome shotgun sequences; see http://www.ncbi.nlm.nih.gov/genomes/lproks.cgi).

We observed a widespread occurrence of putative PKS genes in non-pathogenic clostridia, especially in cellulolytic species, whereas no PKS genes were found in any pathogenic *Clostridium* species. By RT-qPCR we have shown that several PKS coding genes appear to be silent or only basally expressed under standard cultivation conditions, giving a possible explanation as to why the related polyketides have been overlooked so far. The presented data gives an overview of the occurrence and nature of potential PKS genes in non-pathogenic clostridia and provides a foundation for the discovery of novel polyketides from a group of anaerobic bacteria with previously underestimated potential.

## Results

### Genome mining reveals PKS genes exclusively in non-pathogenic *Clostridium* spp

First we aimed at analyzing PKS genes and investigating their relationship with *Clostridium* taxonomy in order to assess their ability to produce various type I PKS metabolites. In this context it should be noted that modular PKS frequently form hybrid assembly-lines with modules of nonribosomal peptide synthetases (NRPS) [8], and thus PKS/NRPS genes were also investigated in this study. To determine the PKS gene distribution in clostridia we analyzed all available genome sequences (both complete and incomplete, published sequencing projects) from the genus *Clostridium* for the occurrence of PKS genes. The module architectures encoded in each gene cluster was annotated using the SEARCHPKS software [9]. Even though nearly 60% of the available genome sequences belong to pathogenic clostridia (six species), we could not detect any PKS genes in these genomes. In contrast, 23% of the remaining non-pathogenic genome sequences (7 out of 31 species) contained a wide variety of bacterial modular type I PKS gene clusters. The solventogenic species *Clostridium acetobutylicum* (CaceA) and *Clostridium thermocellum* (CtheA) feature relatively small putative *cis*-acyltransferase (*cis*-AT) PKS genes, whereas *Clostridium cellulovorans* (CcelvA), *Clostridium beijerinkii* (CbeiA), and *Clostridium kluyveri* (CkluA-C) possess putative hybrid *trans*-AT PKS/NRPS gene clusters (Table 1). These *trans*-acyltransferase PKS modules lack integrated AT domains [10]. By far the most diverse gene cluster assemblies are found in the cellulolytic species *Clostridium cellulolyticum* and *Clostridium papyrosolvens*. *C. cellulolyticum* harbors three putative *trans*-AT hybrid PKS/NRPS gene clusters (CcelA, C, D) as well as one cryptic mixed *cis/trans*-AT hybrid PKS/NRPS gene cluster (CcelB). The *C. papyrosolvens* genome contains even more diverse gene clusters, with five putative *trans*-AT PKS gene clusters (CpapA, C, E, G, H), two *trans*-AT PKS/NRPS gene clusters (CpapB, I), and two mixed *cis/trans*-AT hybrid PKS/NRPS gene clusters (CpapD, F) (Table 1). *Trans*-AT pathways represent promising targets for natural product discovery as they are less well studied and possess unusual enzymatic features that often result in compounds with novel characteristics [11]. The high frequency of these potential PKS genes in clostridia indicates that these organisms could be promising polyketide producers.

**Table 1:** Putative PKS/NRPS gene clusters in sequenced *Clostridium* strains.

*Clostridium* strain	No. putative PKS/NRPS gene clusters	Cluster	Cluster type	Accession No.	No. putative KS domains	KS domains (module structure)
*C. acetobutylicum* ATCC 824	1	CaceA	*cis*-AT PKS	NP_349943-48	1	AKS1
*C. thermocellum* ATCC 27405	1	CtheA	*cis*-AT PKS	YP_001036563-70	2	AKS1-2
*C. cellulovorans* 743B	1	CcelvA	*trans*-AT PKS/NRPS	ZP_04803466-82	1	AKS1
*C. beijerinkii* NCIMB 8052	1	CbeiA	*trans*-AT PKS/NRPS	YP_01307393-403	1	AKS1
*C. kluyveri* DSM 555	3	CkluA	*trans*-AT PKS/NRPS	YP_001395732-47	1	AKS1
		CkluB	*trans*-AT PKS/NRPS	YP_001395118-27	2	BKS1-2
		CkluC	*trans*-AT PKS/NRPS	YP_001394892-919	2	CKS1-2
*C. cellulolyticum* H10	4	CcelA	*trans*-AT PKS/NRPS	YP_002505201-26	16	AKS1-16
		CcelB	*cis/trans*-AT PKS/NRPS	YP_002505303-31	2	BKS1(*trans*-AT)BKS2(*cis*-AT)
		CcelC	*trans*-AT PKS/NRPS	YP_002506639-52	1	CKS1
		CcelD	*trans*-AT PKS/NRPS	YP_002506677-706	3	DKS1-3
*C. papyrosolvens* DSM 2782	9	CpapA	*trans*-AT PKS/NRPS	ZP_05494449-59	2	AKS1-2
		CpapB	*trans*-AT PKS/NRPS	ZP_05494791-801	1	BKS1
		CpapC	*trans*-AT PKS	ZP_05495034-61	12	CKS1-12
		CpapD	*cis/trans*-AT PKS/NRPS	ZP_05495197-219	7	DKS1,3,4 (*cis*-AT); DKS2,5-7 (*trans*-AT)
		CpapE	*trans*-AT PKS	ZP_05496375-85	4	EKS1-4
		CpapF	*cis/trans*-AT PKS/NRPS	ZP_05497951-69	5	FKS1,2 (*cis*-AT); FKS3-5 (*trans*-AT)
		CpapG	*trans*-AT PKS	ZP_05498066-74	3	GKS1-3
		CpapH	*trans*-AT PKS	ZP_05498232-53	3	HKS1-3
		CpapI	*trans*-AT PKS/NRPS	ZP_08192447-65	2	IKS1-2

### Clostridium PKS genes have unusual and diverse phylogenetic origins

**Figure 1:**
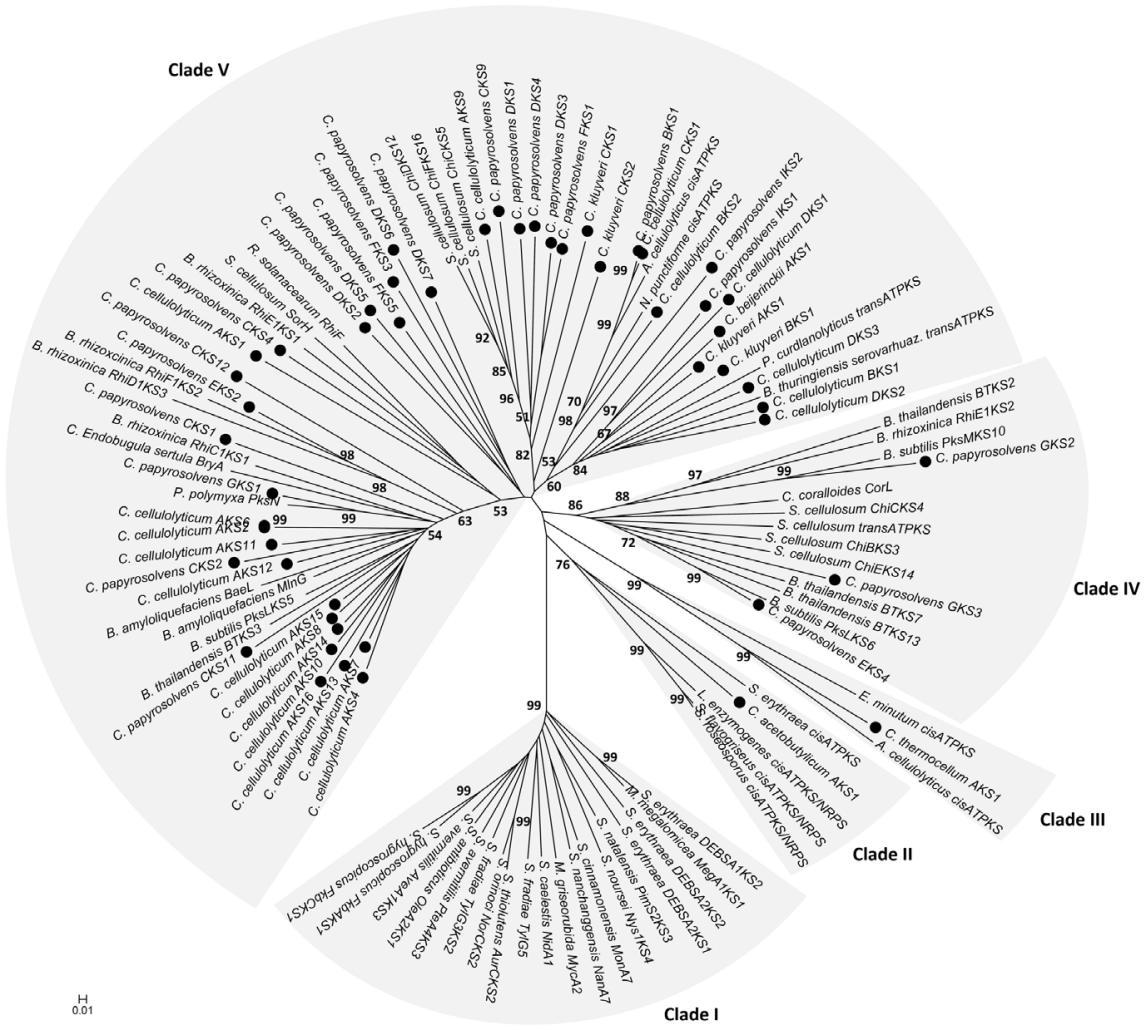
Phylogenetic tree constructed with MEGA 3.1 analysis of KS domain protein sequences from bacterial PKS genes. Bootstrap values above 50% from 1000 re-samplings (neighborhood-joining (NJ) algorithm) are indicated at the nodes. • marks sequences from *Clostridium* species. (List of GenBank accession numbers for KS domains: see table S2).

For further phylogenetic analysis of the identified gene clusters we aligned all annotated KS domain sequences. In earlier studies it has been observed that *cis*-AT KS domains typically cluster tightly in natural product-specific clades, whereas KS domains from *trans*-AT systems cluster according to their substrate type [12]. This results in the distribution of domains from individual pathways among different clades with many clades comprising KS domains from diverse bacterial phyla [12]. Furthermore, KS domains from hybrid PKS/NRPS systems often form distinct clades in phylogenetic trees [2]. It has been suggested that *cis*-AT PKS systems have evolved vertically from free-standing type II fatty acids, while *trans*-AT PKS systems seem to be patchworks acquired from diverse sources and assembled by extensive recombination events and multiple horizontal gene transfers [12]. To investigate the phylogeny of the most highly conserved portions of the clostridial PKS systems, we aligned clostridial KS domains with KS domains from different phyla and PKS types (Figure 1). Surprisingly, the putative *cis*-AT PKS systems of the clostridia do not cluster with the most well-described *cis*-AT PKS systems (clade I), but fall into other clades (clades II to V) (Figure 1). The *cis*-AT PKS systems from *C. thermocellum* and *C. acetobutylicum* align with other unusual *cis*-AT PKS systems in clades II and III. The closest relatives to the KS domain of *C. thermocellum* (clade III) include those from putative *cis*–AT PKS systems within the cellulolytic anaerobe *Acetivibrio cellulolyticus* CD2 and from the gram-negative, strictly anaerobic *Elusimicrobium minutum* Pei191 [13]. It appears that these *cis*-AT PKS from anaerobes form a novel group that evolved separately from the well studied systems within the actinomycetes. The putative *cis*-AT KS domain of *C. acetobutylicum* (clade II) shows homology with KS domains belonging to recently described systems with novel biosynthetic functions, including the cryptic *cis*-AT PKS of *Saccharopolyspora erythraea* NRRL 2338 [14]. The remaining non-canonical potential *cis*-AT hybrid PKS/NRPS systems in this clade II belong to a previously described group of biosynthetic systems conserved among diverse bacteria species for antifungal tetramic acid-containing macrolactams [15]. The HSAF-type PKS/NRPS of *Lysobacter enzymogenes* is a rare example for an assembly line in which a single-module PKS assembles two separate polyketide chains that are then linked together *via* an amide bond of the same amino acid and further modified by tailoring redox enzymes [16]. The putative *trans*-AT hybrid PKS/NRPS gene clusters from the remaining non-pathogenic clostridial species cluster throughout clade V (Figure 1) with all kinds of *trans*-AT PKS and *trans*-AT hybrid PKS/NRPS systems, some with only poor bootstrap values pointing to uncharacterized related polyketides. While the pure *trans*-AT PKS and *trans*-AT hybrid PKS/NRPS systems tend to cluster closer with described *trans*-AT PKSs from *Burkholderia* and *Bacillus* spp. producing e.g. rhizoxin [17], bacillaene [18], or thailandamide [19] the KS domains belonging to putative mixed *cis/trans*-AT hybrid PKS/NRPS gene clusters (CcelB, CpapD, CpapF) are divided into the *trans*-AT KS domains, which cluster with described *trans*-AT PKSs, and the *cis*-AT KS domains, which cluster further apart with other clostridia PKS (Figure 1, Table 1). Taken together, the PKSs from *Clostridium* species appear to have unusual and diverse phylogenetic origins.

### PCR targeting of diverse PKS genes in not yet sequenced *Clostridium* species

Although many genomes of members of the genus *Clostridium* (in particular the human pathogens) have been sequenced, the genomes of many species isolated from a diverse range of habitats are as not yet sequenced. To determine whether some of these not sequenced species also contain putative PKS gene clusters, we designed degenerate oligonucleotide primers to amplify DNA from KS domains within these PKS genes. The primers were designed from the consensus sequences of the aligned putative KS domain sequences from sequenced *Clostridium* spp. Conserved regions with sufficient sequence homology to design *Clostridium* KS-domain-specific primers were identified. The upstream primer was directed against the active-site CSS (5′-TKG AYA CWR YNT GYT CAT C-3′) and the downstream primer was designed to encompass the second active-site histidine HGTGT (5′-TCT CCY AAN GWW GTW CCB GTA CCR TG-3′; product size ∼470 bp). A second degenerate primer pair was designed using the primer encompassing the second active-site HGTGT as upstream primer (5′-CAY GGT ACV GGW ACW WCN TTR GGA GA-3′) and a downstream primer directed to the conserved region FGGG, approximately 115 amino acids downstream of the HGTGT histidine (5′-ATT KGW RCC KCC SRM ACC RAA-3′; product size ∼370 bp). Primer binding sites were distinct from other degenerate primers typically used to screen for KS domains in bacterial genomes. However, due to the homology amongst active site regions within KS domains, correct amplicons were also obtained from *Streptomyces* as well as gram-negative *Burkholderia* spp. harboring type I PKS genes (data not shown). In order to show that these primer sets were able to amplify KS domains from the clostridia, PCR amplification of KS domains from the reference species *C. cellulolyticum* and *C. kluyveri* was performed using both primer sets, and the products were cloned and sequenced. The resulting sequences were identified as known KS domains from these bacteria. In contrast, PCR amplification of DNA from the sequenced *C. butyricum*, which does not possess any putative PKS genes and thus served as a negative control, gave no product (data not shown). The primer sets were then used to screen taxonomically diverse non-sequenced *Clostridium* strains from terrestrial as well as marine environments to get an overview on the occurrence of modular type I PKS genes in non-pathogenic clostridia (Table S1). All chosen *Clostridium* spp. strains were cultured in media recommended from the supplier (DSMZ) under strict anaerobic conditions, and their DNA was isolated. PCR products were cloned and sequenced and the resulting sequences were queried with BLASTX software (http://blast.ncbi.nlm.nih.gov/Blast.cgi)). In some cases different KS domains from one *Clostridium* strain were amplified. Out of fifteen cultivated non-sequenced, non-pathogenic *Clostridium* species three (*C. hungatei*, *C. chartatabidum*, and *C. akagii*) were found to possess putative PKS genes. The deduced sequences of the KS domains showed significant similarity (≥75%) to published and annotated KS domain sequences from established polyketide producers (Table 2).

**Table 2:** BLASTX results of sequences amplified from not yet sequenced *Clostridium* species genomic DNA using KS domain specific degenerated primers.

Amplicon source organism	Clone, Amplicon size (bp); Accession No.	Closest homologue		
		Function (origin)	Accession No.	Protein simililarity/identity (%)
*C. hungatei* DSM 14427	pSB020, 339;HE586558	Beta-ketoacyl synthase (*Bacillus cereus subsp. cytotoxis* NVH 391-98)	YP_001376411	83/65
	pSB021, 339;HE586559	Beta-ketoacyl synthase(*Acetivibrio cellulolyticus CD2*)	ZP_07326740	88/79
	pSB022, 339;HE586560	Polyketide synthase(*Clostridium kluyveri* DSM 555)	YP_001394917	83/72
*C. chartatabidum* DSM 5482	pSB042, 340;HE586561	Polyketide synthase(*Clostridium kluyveri* DSM 555)	YP_001394917	91/80
	pSB043, 386;HE586562	Beta-ketoacyl synthase(*Acetivibrio cellulolyticus* CD2)	ZP_07326740	83/67
*C. akagii* DSM 12554	pSB041, 610;HE586563	Beta-ketoacyl synthase(*Bacillus thuringiensis serovar huazhongensis* BGSC 4BD1)	ZP_04087646	86/78
	pSB044, 335;HE586564	AMP-dependent synthetase and ligase (*Clostridium papyrosolvens* DSM 2782)	ZP_08193005	82/67
	pSB045, 335;HE586565	Beta-ketoacyl synthase(*Clostridium cellulolyticum* H10)	YP_002505214	78/64

Adding these three *Clostridium* species to the ones that we have identified as potential sources for novel polyketides indicates that the frequency of PKS genes in non-pathogenic clostridia may be as high as 20%.

The deduced KS sequence from DNA fragment cloned and sequenced from *C. hungatei* (DSM 14427, isolated from soil under a pile of rotting wood chips; amplified with degenerate primer set two) shows high homology (similarity of 88%) to a *cis*-AT β-ketoacyl synthase from *Acetivibrio cellulolyticus* CD2 and a similarity of 83% to a putative *Bacillus cereus* subsp. *cytotoxicus* NVH 391-98 *trans*-AT beta-ketoacyl synthase (Table 2). The following closest homologues in the BLASTX search are all KS domains from *Clostridium* species (*C. kluyveri*, *C. papyrosolvens* and *C. cellulolyticum*) with similarities >78% to the PCR fragment. A sequence amplified from the genomic DNA of *C. akagii* (DSM 12554, isolated from beech litter) with primer pair one shows significant similarity 86% to a putative KS sequence of *Bacillus thuringiensis serovar huazhongensis* BGSC 4BD1 and 79% similarity with a KS domain of a β-ketoacyl synthase from *C. cellulolyticum* (CcelDKS2). An amplicon from *C. akagii* generated with primer set two shows 82% similarity to a section of a gene from *C. papyrosolvens* annotated as AMP-dependent synthetase and ligase and the next closest homologue (78% similarity) is a potential beta-ketoacyl synthase encoded in the *C. cellulolyticum* genome (CcelAKS13). Furthermore, the deduced KS sequence of the PCR fragment from *C. akagii* is to 78% similar to a KS domain from a putative PKS module encoded in the *Paenibacillus polymyxa* E681 genome (Table 2). A PCR product of *C. chartatabidum* (DSM 5482, isolated from ovine rumen) generated with primer pair two codes for a KS fragment with highest similarity (91%) to the deduced KS from *C. kluyveri* DSM 555 and NBRC 12016 (CkluAKS1), and has significant (83%) similarity to a putative *cis*-AT beta-ketoacyl synthase from *Acetivibrio cellulolyticus* CD2 (Table 2). Taken together, these observations are consistent with the phylogeny of KS domains from sequenced *Clostridium* species, where the novel putative KS domains of non-sequenced species show only limited sequence similarity to well characterized PKS genes of other genera. This further highlights the distinctiveness of the putative polyketide synthases identified within the clostridia and reinforces their potential as producers of diverse polyketides.

### Incongruence of PKS gene occurrence with taxonomy

**Figure 2:**
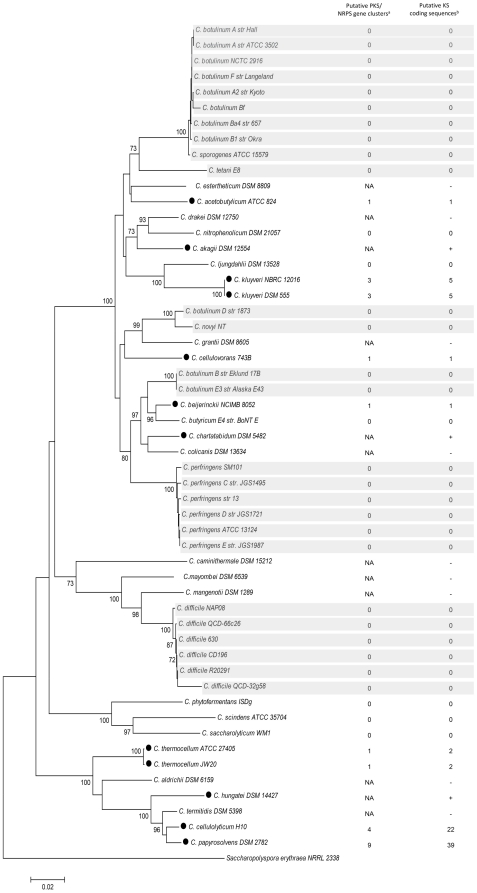
Phylogeny of 16SrDNA sequences of *Clostridium* species. Bootstrap values above 70% from 1000 re-samplings (NJ algorithm) are indicated at the nodes. *Saccharopolyspora erythraea* NRRL 2338 was used as an outgroup for this analysis. Strains shaded in gray are pathogens. Strains marked with • possess PKS genes. NA: not appointed; + indicates new sequenced KS domain sequences; - indicates no positive PCR results. (List of GenBank accession numbers of 16SrDNA sequences: see table S3).

Surprisingly, we observed that potential PKS assembly lines encoded in the genomes of bacteria belonging to the genus *Clostridium* is restricted to non-pathogens. Thus, we aimed at further correlating the distribution and types of PKS genes in clostridia with the phylogeny of the genus. A multiple alignment of 16S ribosomal DNA (16S rDNA) sequences of *Clostridium* spp. strains was used to construct a phylogenetic tree representing clostridial taxonomy (Figure 2). We made a multiple alignment by ClustalW and a phylogenic tree using MEGA version 3.1 with the NJ algorithm [20]. Alignment of the 16S rDNA sequences shows that several separate clades are formed and that there is no distinct separation of pathogens and non-pathogens. *C. difficile* strains, for example, have a larger phylogenetic distance to other pathogenic *Clostridium* species than to non-pathogens. Furthermore, there seems to be little correlation between the evolution of the bacteria and the presence of putative PKS systems. This is shown for the occurrence of putative PKS genes in fully sequenced strains as well as the investigated non-sequenced strains. Particularly noteworthy is the high number of PKS genes in the cellulolytic organisms *C. cellulolyticum* and *C. papyrosolvens*. These results strongly suggest that the clostridia have acquired these PKS gene clusters via horizontal gene transfer independent of strain evolution. This is further supported by the observation that in the proximity of nine out of the twenty identified putative PKS gene clusters in clostridia insertion sequences or other genetic mobility sequences are located. Several potential transposase and/or integrase genes are flanking these gene clusters. This observation correlates well with the hypothesis that *trans*-AT PKS systems are patchworks acquired from diverse sources and assembled by extensive recombination events and multiple horizontal gene transfers [12].

### Cryptic PKS gene clusters are silent under standard cultivation conditions

**Figure 3:**
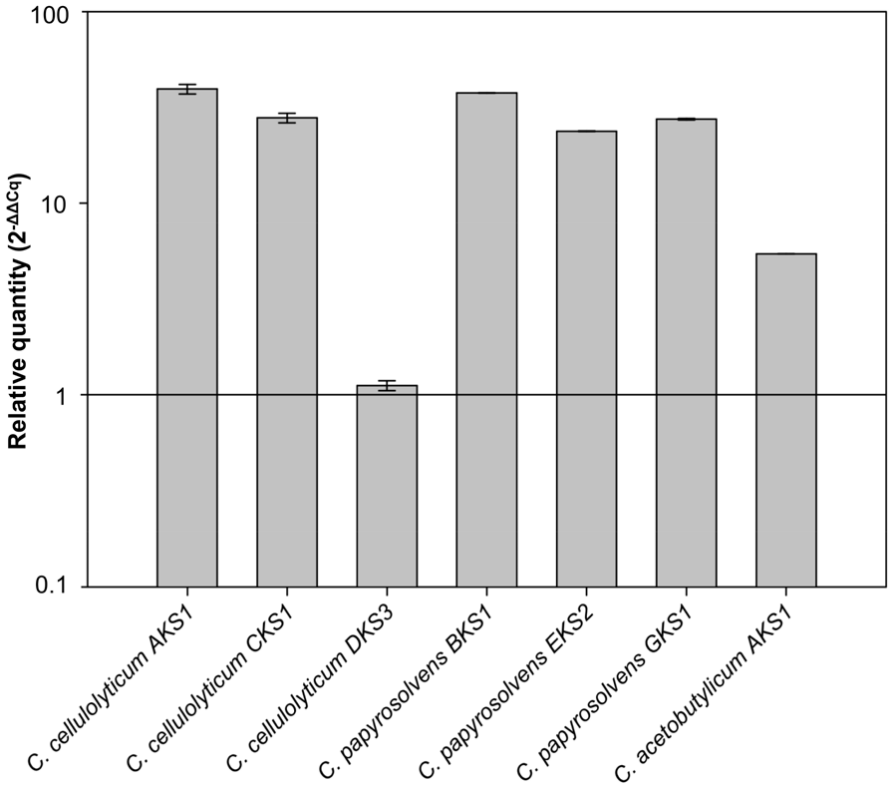
Relative quantity of the mRNA level determined by RT-qPCR of *Clostridium* species PKS genes (KS domains). Relative quantity is given as the log_2_ of −ΔΔCq. Constitiutive expression of the 16S rRNA of each species was used as an internal control and the value of the related non template control of each sample was set to 1 for calibration.

Although the presence of putative PKS and hybrid PKS/NRPS gene clusters within members of the clostridia is indicative of the potential to produce novel compounds, expression of these genes is of course a prerequisite for secondary metabolite production. Thus, we investigated the expression of representative PKS genes (coding for KS domains) from *C. cellulolyticum, C. papyrosolvens*, and *C. acetobutylicum*. Each *Clostridium* spp. strain was cultivated under standard growth conditions and total RNA was isolated from cells harvested in the mid-logarithmic growth phase. PKS gene expression was measured using reverse transcription-quantitative PCR (RT-qPCR) with normalization against 16S rDNA using the expression values of the related non-template controls for calibration [21]. This analysis indicated that in the three organisms tested, PKS gene clusters appear to be silent or only basally expressed under standard laboratory conditions (Figure 3, Figure S1).

## Discussion

*Clostridium* is one of the largest bacteria genera including over 150 metabolically diverse species that occur ubiquitously in soil and in the intestine of higher organisms. The aim of this study was to gain insight into the potential of anaerobic *Clostridium* spp. to produce polyketide metabolites. We analyzed all available *Clostridium* genome sequences and found that the presence of putative PKS genes is clearly restricted to non-pathogenic organisms. In total, 23% of the non-pathogenic genome sequences (7 out of 31 species) harbored diverse modular type I PKS gene clusters. This frequency is supported by PCR screening of fifteen cultivated non-sequenced, non-pathogenic *Clostridium* species, out of which three possess type I PKS genes. Interestingly, a phylogenetic analysis revealed that *Clostridium* PKS genes have unusual and diverse phylogenetic origins. We not only detected putative *cis*- and *trans*-AT PKS genes, but also genes for unusual types of modular PKS, which fall into novel clades in the phylogenetic tree. Despite the exclusive distribution of PKS genes in non-pathogenic clostridia, the occurrence of PKS genes is not congruent with taxonomic relationships. Thus, it is plausible that the PKS gene clusters have evolved independent of strain evolution, and their diversity points to diverse sources involving extensive recombination events and multiple horizontal gene transfers. The finding that the *Clostridium* PKS systems apparently have unusual and diverse phylogenetic origins also suggests that the pathways encoded lead to diverse polyketide metabolites. However, we found that all hitherto investigated cryptic PKS gene clusters are silent under standard cultivation conditions. As has been shown for *C. cellulolyticum*, particular signaling compounds, nutrients or other unknown environmental triggers are be needed to activate the apparently silent closthioamide biosynthesis genes [6]. This phenomenon is well known for other metabolic pathways in bacteria and fungi [22] and is plausible in an anaerobic world where energy is a rare commodity. The dormant state of these putative PKS gene clusters might also explain why the related metabolites have been overlooked until now. Whereas the high frequency of PKS genes in non-pathogenic clostridia indicates that these organisms could serve as promising polyketide sources, the on-going challenge is to understand the regulatory mechanisms and to find ways to activate gene expression. This may either involve genetic engineering including heterologous expression or applying particular stimuli for inducing gene cluster expression in the wild type. It stands to reason that non-pathogenic clostridia may use more than just the ability to live in anaerobic niches to be as successful as they are in their habitats and that the chemical weapons they might have developed could be novel bioactive molecules. The recent discovery of the first antibiotic from the strictly anaerobic world, closthioamide, encourages future research in this direction [6]. Furthermore, we introduced degenerate primer pairs that can be used to screen further non-pathogenic *Clostridium* species for more potential PKS genes. Taken together, this study provides an insight into the distribution and diversity of PKS genes in non-pathogenic clostridia and facilitates the discovery of novel polyketides from a group of strict anaerobic bacteria with previously unexplored potential.

## Materials and Methods

### Bacterial strains and culture conditions

Table S1 contains a list of each of the strains utilized in this study. Strains were obtained from DSMZ GmbH (Braunschweig) as stock cultures and grown anaerobically in serum glass bottles sealed with butyl rubber stoppers and aluminium crimps in media derived from recommended media of the DSMZ GmbH at the appropriate temperature without shaking. *Clostridium cellulolyticum* (DSM 5812) (37°C), *C. phytofermentas* (DSM 18823) (30°C), *C. aldrichii* (DSM 6159) (37°C): Medium CM3 modified as described by Lincke et al. [6] *C. kluyveri* (DSM 555) (30°C): Medium M52 with Na_2_S×9H_2_O replaced by cysteine-HCl×H_2_O and a pH of 7.0. *C. isatidis* (DSM 15098) (37°C), *C. mangenotii* (DSM 1289) (26°C), *C. oceanicum* (DSM 1290) (30°C), *C. chartatabidum* (DSM 5482) (37°C): Medium M110 with ground beef solution replaced by beef extract (desiccated, Difco) 5 g/L plus haemin and vitamin K1 solution. *C. thermocellum* (DSM 1237) (30°C): Medium M122 with cellobiose 5.0 g L^−1^ and pH adjusted to 7.1. *C. acetobutylicum* (DSM) 792 (37°C), *C. butyricum* (DSM 10702) (37°C), *C. drakei* (DSM 12750) (30°C), *C. nitrophenolicum* (DSM 21057) (30°C): Medium M104b. *C. papyrosolvens* (DSM 2782) (37°C): Medium M289 with sea water replaced by instant ocean (Aquarium systems) 6.67 g L^−1^ and cellobiose 5.0 g L^−1^ plus cellulose (MN 300) 3.0 g L^−1^ and addition of reinforced clostridial medium (RCM) (Difco) 19 g L^−1^. *C. akagii* (DSM 12554) (26°C): Medium M869. *C. camnithermale* (DSM 15212) (42°C): Medium M986 with instant ocean (Aquarium systems) 33.3 g L^−1^ and Na_2_S×9 H_2_O replaced by cysteine-HCl×H_2_O. *C. colicanis* (DSM 13634) (37°C): Medium M104 with beef extract (desiccated, Difco) 5.0 g L^−1^. *C. estertheticum* (DSM 8809) (10°C): Medium M642 with RCM (Difco) 38 g L^−1^. *C. grantii* (DSM 8605) (30°C): Medium M648 with Na_2_S×9 H_2_O replaced by cysteine-HCl×H_2_O. *C. mayombei* (DSM 6539) (30°C): Medium M515. *C. termitidis* (DSM 5398) (37°C): Medium M539 with cellobiose 5.0 g L^−1^ and Na_2_S×9 H_2_O replaced by cysteine-HCl×H_2_O. *C. hungatei* (DSM 14427) (30°C): Medium M255.

### Isolation of total RNA and gene expression analysis

Total RNA was isolated from 50 mL of culture of *C. cellulolyticum* DSM 5812, *C. papyrosolvens* DSM 2782 and *C. acetobulylicum* DSM 792, respectively. RNAprotect Bacteria Reagent (Qiagen GmbH, Hilden) was quickly added to the samples and cell pellets were harvested following the manufacturer's protocol, and stored at −20°C. For RNA isolation, cells were resuspended in TE buffer (30 mM Tris-HCl, 1 mM EDTA, pH 8.0) containing 15 mg mL^−1^ lysozyme (1 volume), vortexed for 10 s and incubated at room temperature for 1 h. RNA was purified using RNeasy Mini Kit (Qiagen GmbH, Hilden, Germany) following the manufacturer's protocol and afterwards stored at −80°C. DNA was removed by digestion utilizing the TURBO DNA-free Kit (Applied Biosystems, Ambion Inc., Austin, USA) following the manufacturer's protocol with incubation for 40–90 min at 37°C. RT-qPCR experiments were performed when RNA samples were confirmed to be DNA free, as indicated by a lack of product formation in a PCR amplification using DNA polymerase and the RT-PCR primers on an RNA template. RNA quantity was measured in a spectrophotometer (NanoDrop, ND-1000; PeQLab Biotechnologie GmbH, Erlangen). One-step reverse transcription-quantitative PCRs were performed using a One Step SYBR PrimeScript RT-PCR Kit (TAKARA Bio Inc., Otsu Shiga, Japan). 100 ng of RNA were applied to each RT-qPCR reaction mixture. 16S rRNA gene of each species was used as an internal standard for calculation of expression levels. (Primer pairs: *C. cellulolyticum*: AKS1: CcelA1RT_f: GCT GAC GGA AGA TGC AAG AC and CcelA1RT_r: TCC AGA GGC TGA AGC AAC AA; CKS1: CcelC1RT_f: ATC AGG CTC CAC AGG TAA GC and CcelC1RT_r: CGC AAT AGA CCA CCA GAA CA; 16S rRNA: Ccel16SRT_f: GAC GAC AAC CAT GCA CCA C and Ccel16SRT_r: CAA CGC GAA GAA CCT TAC CA; DKS3: CcelD3RT_f: CGC ATT CTC CGT TCC TTA TTG and CcelD3RT_r: TGC ATG TTC CTC ATC ACT GG; *C. acetobutylicum*: AKS1: CaceA1RT_f: TCT TGG GAA GCC TTT GAG GA and CaceA1RT_r: TCT CCT GTG GCT GAC TGT GAT T; 16S rRNA: Cace16SRT_f: GCG GTG AAA TGC GTA GAG AT and Cace16SRT_r: ACT TCC CAG GCG GAA TAC TT; *C. papyrosolvens*: BKS1: CpapB1RT_f: GCG GAC TTG GCA TAT TAG CA and CpapB1RT_r: CCC AGA TTT GTA CCC GTT CC; EKS2: CpapE2RT_f: ATG TCG GCG CAA TGT ATC AG and CpapE2RT_r: TGG CAG AAG AAG AAC AAG CAG; GKS1: CpapG1RT_f: GAG CTT GCG GAA TGG AAG AG and CpapG1RT_r: CGG ACA GCA CAA CAA TAG CC; 16S rRNA: Cpap16SRT_f: GCG GTG AAA TGC GTA GAT AT and Cpap16SRT_r: GTT AAC TCC GGC ACA GAA GG). For negative controls, experiments were performed using water instead of RNA, and for non-template controls without RT enzyme mix, respectively. The reactions were executed in an Eppendorf realplex^2^ mastercycler (epgradientS, Eppendorf AG, Hamburg) in triplicate for each sample. Cycling parameters included an initial DNA denaturation step at 95°C for 2 min, followed by 40 cycles with DNA denaturation at 95°C for 5 s, primer annealing at 60°C for 15 s, and elongation at 72°C for 10 s. Relative expression levels for each mRNA sample were obtained using the ΔΔCq method via normalization to 16S rRNA applying the formula 2^−(ΔCq,q- ΔCq,cb)^ for all samples. With ΔC_q,q_ = C_q,PKS target_−C_q,16SrRNA_ and ΔC_q,cb_ = C_q,calibrator_−C_q,16SrRNA_. The expression values of the related non template controls of each sample were used for calibration (maximum value: C_q,calibrator_ = 40). The appendent value of the calibrator is set as 1 (2^−(ΔCq,cb- ΔCq,cb)^ = 1) [21].

### Isolation of genomic DNA

1.5 mL of cultured bacterial cells were centrifuged and the pelleted cells were resuspended in TE buffer and used for isolation of genomic DNA with MasterPure™ Gram Positive DNA Purification Kit (Epicentre Biotechnologies, Madison, Wisconsin, USA). The precipitated DNA was finally resuspended in 20 µL TE buffer and directly used for PCR analysis.

### Design of degenerate KS domain-specific primers

27 putative *Clostridium* KS domains were annotated using SEARCHPKS software [9] and a multiple alignment was performed using ClustalW [20]. Highly conserved regions among *Clostridium* KS-domain were identified as “VDTMCSS”, “HGTGTSLGD” and “FGGGSN” and these sequences were used to design KS-domain-specific primers. From these degenerated nucleic acid sequences were evolved with a maximum degeneration grade (dg) of 384. Two resulting primer sets were used to amplify DNA encoding KS domains, respectively: pair 1: degKS_1_f: 5′-TKG AYA CWR YNT GYT CAT C-3′ (18 bp, dg 256), degKS_3_r: 5′-TCT CCY AAN GWW GTW CCB GTA CCR TG-3′ (24 bp, dg 384); product size approx. 470 bp and pair 2: degKS_3_f: 5′-CAY GGT ACV GGW ACW WCN TTR GGA GA-3′ (24 bp, dg 384), degKS_5_r: 5′-ATT KGW RCC KCC SRM ACC RAA-3′ (21 bp, dg 256); product size approx. 370 bp. PCR conditions: 100 ng gDNA; 100 pmol primer; program: 94°C 5 min, 94°C 15 sec, 42°C 30 sec, 72°C 1 min, 33×, 72°C 2 min, 4°C hold. The PCR products were cloned into pGEM®T-Easy Vector (Promega Corporation) or pCR®2.1-TOPO® vector (TOPO TA Cloning Kit®, Invitrogen) following the manufacturers protocol, amplified in *E. coli* and sequenced by Eurofins MWG. The resulting sequences have been deposited in GenBank (Accession No. HE586558 - HE586565) and were analyzed using BLASTX software (http://blast.ncbi.nlm.nih.gov/Blast.cgi) Significant similarities (>75%) to published sequences were analyzed.

## Supporting Information

Figure S1 **Relative quantity of the mRNA level determined by RT-qPCR of** ***Clostridium*** **species PKS genes at different standard cultivation time points (early exponential – stationary growth phase).**
(PDF)

Table S1 **Cultivated** ***Clostridium*** **species and the habitat they were isolated from.**
(PDF)

Table S2 **List of GenBank accession numbers for KS domain sequences.**
(PDF)

Table S3 **List of GenBank accession numbers of 16SrDNA sequences.**
(PDF)

## References

[1] Hertweck C (2009). The biosynthetic logic of polyketide diversity.. Angew Chem Int Ed.

[2] Jenke-Kodama H, Sandmann A, Muller R, Dittmann E (2005). Evolutionary implications of bacterial polyketide synthases.. Mol Biol Evol.

[3] Winter JM, Behnken S, Hertweck C (2011). Genomics-inspired discovery of natural products.. Curr Opin Chem Biol.

[4] Metsä-Ketelä M, Halo L, Munukka E, Hakala J, Mäntsälä P (2002). Molecular evolution of aromatic polyketides and comparative sequence analysis of polyketide ketosynthase and 16S ribosomal DNA genes from various *Streptomyces* species.. Appl Environ Microbiol.

[5] Jenke-Kodama H, Borner T, Dittmann E (2006). Natural biocombinatorics in the polyketide synthase genes of the actinobacterium *Streptomyces avermitilis*.. PLoS Comput Biol.

[6] Lincke T, Behnken S, Ishida K, Roth M, Hertweck C (2010). Closthioamide: An unprecedented polythioamide antibiotic from the strictly anaerobic bacterium *Clostridium cellulolyticum*.. Angew Chem Int Ed.

[7] Paredes CJ, Alasker KV, Papoutsakis ET (2005). A comparative genomic view of clostridial sporulation and physiology.. Nat Rev Microbiol.

[8] Du L, Sanchez C, Shen B (2001). Hybrid peptide-polyketide natural products: biosynthesis and prospects toward engineering novel molecules.. Metab Eng.

[9] Yadav G, Gokhale RS, Mohanty D SEARCHPKS.

[10] Piel J Biosynthesis of polyketides by *trans*-AT polyketide synthases.. Nat Prod Rep.

[11] Teta R, Gurgui M, Helfrich EJ, Kunne S, Schneider A (2010). Genome mining reveals trans-AT polyketide synthase directed antibiotic biosynthesis in the bacterial phylum bacteroidetes.. ChemBioChem.

[12] Nguyen T, Ishida K, Jenke-Kodama H, Dittmann E, Gurgui C (2008). Exploiting the mosaic structure of trans-acyltransferase polyketide synthases for natural product discovery and pathway dissection.. Nat Biotechnol.

[13] Herlemann DPR, Geissinger O, Ikeda-Ohtsubo W, Kunin V, Sun H (2009). Genomic Analysis of “*Elusimicrobium minutum*”, the first cultivated representative of the phylum “*Elusimicrobia*” (Formerly Termite Group 1).. Appl Environ Microbiol.

[14] Boakes S, Oliynyk M, Cortes J, Bohm I, Rudd BA (2004). A new modular polyketide synthase in the erythromycin producer *Saccharopolyspora erythraea*.. J Mol Microbiol Biotechnol.

[15] Lou L, Qian G, Xie Y, Hang J, Chen H (2011). Biosynthesis of HSAF, a tetramic acid-containing macrolactam from *Lysobacter enzymogenes*.. J Am Chem Soc.

[16] Blodgett JA, Oh DC, Cao S, Currie CR, Kolter R (2010). Common biosynthetic origins for polycyclic tetramate macrolactams from phylogenetically diverse bacteria.. Proc Natl Acad Sci.

[17] Partida-Martinez L, Hertweck C (2007). A gene cluster encoding rhizoxin biosynthesis in “Burkholderia rhizoxina”, the bacterial endosymbiont of the fungus *Rhizopus microsporus*.. Chem Bio Chem.

[18] Moldenhauer J, Xiao-Hua C, Borriss R, Piel J (2007). Biosynthesis of the antibiotic bacillaene, the product of a giant polyketide synthase complex of the *trans*-AT family.. Angew Chem Int Ed.

[19] Ishida K, Lincke T, Behnken S, Hertweck C (2010). Induced biosynthesis of cryptic polyketide metabolites in a *Burkholderia thailandensis* quorum sensing mutant.. J Am Chem Soc.

[20] Kumar S, Tamura K, Nei M (2004). MEGA3: Integrated software for molecular evolutionary genetics analysis and sequence alignment.. Briefings in Bioinformatics.

[21] Livak KJ, Schmittgen TD (2001). Analysis of relative gene expression data using real-time quantitative PCR and the 2^−ΔΔCt^ method.. Methods.

[22] Scherlach K, Hertweck C (2009). Triggering cryptic natural product biosynthesis in microorganisms.. Org Biomol Chem.

