# Supplementary material for: Cryptic Polyketide Synthase Genes in Non-Pathogenic Clostridium SPP
Source: PLoS One. 2012 Jan 3;7(1):e29609. doi: 10.1371/journal.pone.0029609 (PMC3250452; doi:10.1371/journal.pone.0029609)

Figure S1. Relative quantity of the mRNA level determined by RT-qPCR of *Clostridium* species PKS genes (KS domains) at different standard cultivation time points (early exponential – stationary growth phase). Relative quantity is given as the $\log_2$ of $-\Delta\Delta C_q$. Constitutive expression of the 16S rRNA of each species was used as an internal control and C_q values of non-template controls of each sample where used for calibration ($2^{-\Delta\Delta C_{q,cb}} = 1$); A: *C. acetobutylicum* AKS1; B: *C. papyrosolvens* EKS2.

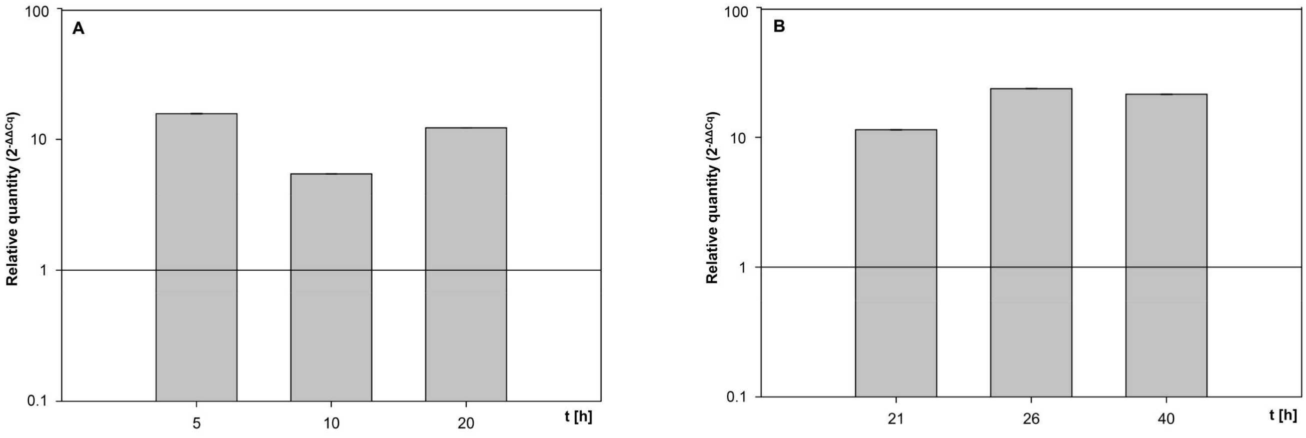

Supplement: Figure S1 — Relative quantity of the mRNA level determined by RT-qPCR of Clostridium species PKS genes at different standard cultivation time points (early exponential – stationary growth phase). (PDF) [file pone.0029609.s001.pdf]
