# Supplementary material for: Cryptic Polyketide Synthase Genes in Non-Pathogenic Clostridium SPP
Source: PLoS One. 2012 Jan 3;7(1):e29609. doi: 10.1371/journal.pone.0029609 (PMC3250452; doi:10.1371/journal.pone.0029609)
Supplement: Table S1 — Cultivated Clostridium species and the habitat they were isolated from. (PDF) [file pone.0029609.s002.pdf]

Table S1. Cultivated *Clostridium* species and the habitat they were isolated from.

***Clostridium* spp.**	**Strain**	**Natural habitat**
C. acetobutylicum	DSM 792	Soil
C. akagii	DSM 12554	Beech litter
C. aldrichii	DSM 6159	Wood-fermenting anaerobic digestor
C. butyricum	DSM 10702	Intestine of pig
C. caminithermale	DSM 15212	Deep-sea hydrothermal vent.
C. cellulolyticum	DSM 5812	Decayed grass compost
C. chartatabidum	DSM 5482	Ovine rumen
C. colicanis	DSM 13634	Canine individual (gut bacterial)
C. drakei	DSM 12750	Acidic coal mine pond
C. estertheticum	DSM 8809	Vacuum packed beef
C. grantii	DSM 8605	Mullet gut
C. hungatei	DSM 14427	Soil under a pile of rotting wood chips
C. isatidis	DSM 15098	Woad vat
C. kluyveri	DSM 555	Mud
C. mangenotii	DSM 1289	Soil
C. mayombei	DSM 6539	Gut, termite *Cubitermes* sp.
C. nitrophenolicum	DSM 21057	Subsurface soil sample
C. oceanicum	DSM 1290	Marine sediment
C. papyrosolvens	DSM 2782	Paper-mill effluent
C. phytofermentas	DSM 18823	Forest soil
C. termitidis	DSM 5398	Hindgut, termite *Nasutitermes lujae*
C. thermocellum	DSM 1237	Horse manure
