# Supplementary material for: Cryptic Polyketide Synthase Genes in Non-Pathogenic Clostridium SPP
Source: PLoS One. 2012 Jan 3;7(1):e29609. doi: 10.1371/journal.pone.0029609 (PMC3250452; doi:10.1371/journal.pone.0029609)
Supplement: Table S2 — List of GenBank accession numbers for KS domain sequences. (PDF) [file pone.0029609.s003.pdf]

Table S2. List of GenBank accession numbers for KS domain sequences.

Organism	Cluster type	KS domain	Accession No.
**Clade I**			
Saccharopolyspora erythraea NRRL 2338	*cis*-AT PKS	DEBSA1KS2	ZP_06567595
Micromonospora megalomicea subsp. nigra	*cis*-AT PKS	MegA1KS1	AAG13917
Saccharopolyspora erythraea NRRL 2338	*cis*-AT PKS	DEBSA2KS2	YP_001102990
Saccharopolyspora erythraea NRRL 2338	*cis*-AT PKS	DEBSA2KS1	YP_001102990
Streptomyces natalensis	*cis*-AT PKS	PimS2KS3	CAC20921
Streptomyces noursei ATCC 11455	*cis*-AT PKS	NysIKS4	AAF71766
Streptomyces cinnamonensis	*cis*-AT PKS	MonA7	AAO65807
Streptomyces nanchangensis	*cis*-AT PKS	NanA7	AAP42867
Micromonospora griseorubida	*cis*-AT PKS	MycA2	BAC57029
Streptomyces caelestis	*cis*-AT PKS	NidA1	AAC46024
Streptomyces fradiae	*cis*-AT PKS	TylG5	AAB66508
Streptomyces thioluteus	*cis*-AT PKS	AurCKS2	CAE02606
Streptomyces orinoci	*cis*-AT PKS	NorCKS2	CAO85898
Streptomyces fradiae	*cis*-AT PKS	TylG3KS2	AAB66506
Streptomyces avermitilis MA-4680	*cis*-AT PKS	PteA4KS3	NP_821591
Streptomyces antibioticus	*cis*-AT PKS	OleA2KS3	AAF82409
Streptomyces avermitilis	*cis*-AT PKS	AveA1KS3	AAG09812
Streptomyces hygroscopicus subsp. ascomyceticus	*cis*-AT PKS	FkbAKS1	AAF86396
Streptomyces hygroscopicus subsp. ascomyceticus	*cis*-AT PKS	FkbCKS1	AAF86392
**Clade II**			
Saccaharopolyspora erythraea NRRL 2338	*cis*-AT PKS	-	YP_001104811
Clostridium acetobutylicum ATCC 824	*cis*-AT PKS	AKS1	NP_349947
Lysobacter enzymogenes	*cis*-AT PKS/NRPS	-	ABL86391
Streptomyces flavogriseus ATCC 33331	*cis*-AT PKS/NRPS	-	ZP_05802686
Streptomyces roseosporus NRRL 11379	*cis*-AT PKS/NRPS	-	ZP_04712879
**Clade III**			
Elusimicrobium minutum Pei191	*cis*-AT PKS	-	YP_001875990
Clostridium thermocellum ATCC 27405	*cis*-AT PKS	AKS1	YP_001036569
Acetivibrio cellulolyticus CD2	*cis*-AT PKS	-	ZP_07327677
**Clade IV**			
Burkholderia thailandensis E264	*trans*-AT PKS/NRPS	BTKS2	ZP_05591284
Burkholderia rhizoxinica HKI 454	*trans*-AT PKS/NRPS	RhiE1KS2	YP_004029393
Bacillus subtilis subsp. subtilis str. 168	*trans*-AT PKS/NRPS	PksMKS10	ZP_03591444
Clostridium papyrosolvens DSM 2782	*trans*-AT PKS	GKS2	ZP_05498070
Corallococcus coralloides	*trans*-AT PKS	CorL	ADI59534
Sorangium cellulosum 'So ce 56'	*trans*-AT PKS/NRPS	ChiCKS4	YP_001614779

Sorangium cellulosum 'So ce 56'	*trans*-AT PKS	-	YP_001613827
Sorangium cellulosum 'So ce 56'	*trans*-AT PKS/NRPS	ChiCBKS3	YP_001614780
Sorangium cellulosum 'So ce 56'	*trans*-AT PKS/NRPS	ChiEKS14	YP_001614777
Clostridium papyrosolvens DSM 2782	*trans*-AT PKS	GKS3	ZP_05498070
Burkholderia thailandensis E264	*trans*-AT PKS/NRPS	BTKS7	YP_439861
Burkholderia thailandensis E264	*trans*-AT PKS/NRPS	BTKS13	YP_439859
Bacillus subtilis subsp. subtilis str. 168	*trans*-AT PKS/NRPS	PksLKS6	ZP_03591443
Clostridium papyrosolvens DSM 2782	*trans*-AT PKS	EKS4	ZP_05496379
**Clade V**			
Clostridium cellulolyticum H10	*trans*-AT PKS/NRPS	AKS4	YP_002505210
Clostridium cellulolyticum H10	*trans*-AT PKS/NRPS	AKS7	YP_002505211
Clostridium cellulolyticum H10	*trans*-AT PKS/NRPS	AKS13	YP_002505213
Clostridium cellulolyticum H10	*trans*-AT PKS/NRPS	AKS16	YP_002505214
Clostridium cellulolyticum H10	*trans*-AT PKS/NRPS	AKS10	YP_002505212
Clostridium cellulolyticum H10	*trans*-AT PKS/NRPS	AKS14	YP_002505213
Clostridium cellulolyticum H10	*trans*-AT PKS/NRPS	AKS8	YP_002505211
Clostridium cellulolyticum H10	*trans*-AT PKS/NRPS	AKS15	YP_002505213
Clostridium papyrosolvens DSM 2782	*trans*-AT PKS	CKS11	ZP_05495051
Burkholderia thailandensis E264	*trans*-AT PKS/NRPS	BTKS3	YP_439868
Bacillus subtilis subsp. subtilis str. 168	*trans*-AT PKS/NRPS	PksLKS5	ZP_03591443
Bacillus amyloliquefaciens FZB42	*trans*-AT PKS/NRPS	MlnG	YP_001421033
Bacillus amyloliquefaciens FZB42	*trans*-AT PKS/NRPS	BaeL	YP_001421293
Clostridium cellulolyticum H10	*trans*-AT PKS/NRPS	AKS12	YP_002505213
Clostridium papyrosolvens DSM 2782	*trans*-AT PKS	CKS2	ZP_05495042
Clostridium cellulolyticum H10	*trans*-AT PKS/NRPS	AKS11	YP_002505212
Clostridium cellulolyticum H10	*trans*-AT PKS/NRPS	AKS2	YP_002505209
Clostridium cellulolyticum H10	*trans*-AT PKS/NRPS	AKS6	YP_002505210
Paenibacillus polymyxa SC2	*trans*-AT PKS/NRPS	PksN	YP_003947587
Clostridium papyrosolvens DSM 2782	*trans*-AT PKS	GKS1	ZP_05498069
Candidatus Endobugula sertula	*trans*-AT PKS/NRPS	BryA	ABM63537
Burkholderia rhizoxinica HKI 454	*trans*-AT PKS/NRPS	RhiC1KS1	YP_004029396

Clostridium papyrosolvens DSM 2782	*trans*-AT PKS	CKS1	ZP_05495042
Burkholderia rhizoxinica HKI 454	*trans*-AT PKS/NRPS	RhiD1KS3	YP_004029395
Burkholderia rhizoxinica HKI 454	*trans*-AT PKS/NRPS	RhiF1KS2	YP_004029392
Clostridium papyrosolvens DSM 2782	*trans*-AT PKS	EKS2	ZP_05496377
Clostridium papyrosolvens DSM 2782	*trans*-AT PKS	CKS12	ZP_05495052
Clostridium cellulolyticum H10	*trans*-AT PKS/NRPS	AKS1	YP_002505209
Clostridium papyrosolvens DSM 2782	*trans*-AT PKS	CKS4	ZP_05495043
Burkholderia rhizoxinica HKI 454	*trans*-AT PKS/NRPS	RhiE1KS1	YP_004029393
Sorangium cellulosum	*trans*-AT PKS/NRPS	SorH	ADN68483
Ralstonia solanacearum CFBP2957	*trans*-AT PKS/NRPS	RhiF	YP_003748162
Clostridium papyrosolvens DSM 2782	*cis/trans*-AT PKS/NRPS	DKS2	ZP_05495197
Clostridium papyrosolvens DSM 2782	*cis/trans*-AT PKS/NRPS	DKS5	ZP_05495198
Clostridium papyrosolvens DSM 2782	*cis/trans*-AT PKS/NRPS	FKS5	ZP_05497962
Clostridium papyrosolvens DSM 2782	*cis/trans*-AT PKS/NRPS	FKS3	ZP_05497960
Clostridium papyrosolvens DSM 2782	*cis/trans*-AT PKS/NRPS	DKS6	ZP_05495201
Clostridium papyrosolvens DSM 2782	*cis/trans*-AT PKS/NRPS	DKS7	ZP_05495201
Sorangium cellulosum 'So ce 56'	*trans*-AT PKS/NRPS	ChiDKS12	YP_001614778
Sorangium cellulosum 'So ce 56'	*trans*-AT PKS/NRPS	ChiFKS16	YP_001614776
Sorangium cellulosum 'So ce 56'	*trans*-AT PKS/NRPS	ChiCKS5	YP_001614779
Clostridium cellulolyticum H10	*trans*-AT PKS/NRPS	AKS9	YP_002505211
Clostridium papyrosolvens DSM 2782	*trans*-AT PKS	CKS9	ZP_05495049
Clostridium papyrosolvens DSM 2782	*cis/trans*-AT PKS/NRPS	DKS1	ZP_05495197
Clostridium papyrosolvens DSM 2782	*cis/trans*-AT PKS/NRPS	DKS4	ZP_05495198
Clostridium papyrosolvens DSM 2782	*cis/trans*-AT PKS/NRPS	DKS3	ZP_05495197
Clostridium papyrosolvens DSM 2782	*cis/trans*-AT PKS/NRPS	FKS1	ZP_05497959
Clostridium kluyveri DSM 555	*trans*-AT PKS/NRPS	CKS1	YP_001394917
Clostridium kluyveri DSM 555	*trans*-AT PKS/NRPS	CKS	YP_001394917
Clostridium papyrosolvens DSM 2782	*trans*-AT PKS/NRPS	BKS1	ZP_05494798
Clostridium cellulolyticum H10	*trans*-AT PKS/NRPS	CKS1	YP_002506645
Acetivibrio cellulolyticus CD2	*cis*-AT PKS	-	ZP_07328050
Nostoc punctiforme PCC 73102	*cis*-AT PKS	-	YP_001866780

Clostridium cellulolyticum H10	*trans*-AT PKS/NRPS	BKS2	YP_002505319
Clostridium papyrosolvens DSM 2782	*trans*-AT PKS/NRPS	IKS2	ZP_08192452
Clostridium papyrosolvens DSM 2782	*trans*-AT PKS/NRPS	IKS1	ZP_08192449
Clostridium cellulolyticum H10	*trans*-AT PKS/NRPS	DKS1	YP_002506688
Clostridium beijerinkii NCIMB 8052	*trans*-AT PKS/NRPS	AKS1	YP_001307397
Clostridium kluyveri DSM 555	*trans*-AT PKS/NRPS	AKS1	YP_001395738
Clostridium kluyveri DSM 555	*trans*-AT PKS/NRPS	BKS1	YP_001395121
Paenibacillus curdlanolyticus YK9	*trans*-AT PKS	-	ZP_07390016
Clostridium cellulolyticum H10	*trans*-AT PKS/NRPS	DKS3	YP_002506697
Bacillus thuringiensis serovar huazhongensis BGSC 4BD1	*trans*-AT PKS	-	ZP_04087646
Clostridium cellulolyticum H10	*trans*-AT PKS/NRPS	BKS1	YP_002505319
Clostridium cellulolyticum H10	*trans*-AT PKS/NRPS	DKS2	YP_002506689
