# Supplementary material for: Cryptic Polyketide Synthase Genes in Non-Pathogenic Clostridium SPP
Source: PLoS One. 2012 Jan 3;7(1):e29609. doi: 10.1371/journal.pone.0029609 (PMC3250452; doi:10.1371/journal.pone.0029609)
Supplement: Table S3 — List of GenBank accession numbers of 16SrDNA sequences. (PDF) [file pone.0029609.s004.pdf]

Table S3. List of GenBank accession numbers of 16SrDNA sequences.

Species	Strain	16S Gene ID
Clostridium botulinum	Ba4 str. 657	7878427
	B str. Eklund 17B	6294381
	B1 str. Okra	6150965
	A2 str. Kyoto	7764219
	A str. Hall	5399263
	A str. ATCC 19397	5395166
	A str. ATCC 3502	5187397
	E3 str. Alaska E43	6318394
	E1 str. BoNT E Beluga	Pathema:CLO_0001
	F str. Langeland	5402200
	D str. 1873	Pathema:CLG_B0479
	C str. Eklund	CBC_1123
	Bf	Pathema:CBB_4060
	NCTC 2916	CBN_3796
Clostridium cellulolyticum	H10	7312098
Clostridium kluyveri	NBRC 12016	7275018
	DSM 555	5393192
Clostridium phytofermentas	ISDg	5741490
Clostridium beijerinckii	NCIMB 8052	5291242
Clostridium difficile	630	4916094
	QCD-66c26	CdifQC_020100r0057
	QCD-32g58	CdifQ_040500r02145
	R20291	8470276
	NAP08	HMPREF0220_r0001
	CD 196	8466630
Clostridium thermocellum	ATCC 27405	4808996
	DSM 2360	ClothDRAFT_R0054
Clostridium novyi	NT	4541522
Clostridium perfringens	SM101	4794288
	ATCC 13124	4202954
	str. 13	988236
	D str. JGS1721	CJD_0317
	E str. JGS1987	AC3_2841
	C str. JGS1495	CPC_A0100
Clostridium tetani	E88 chromosome	2732761
Clostridium acetobutylicum	ATCC 824	3135244
Clostridium papyrosolvens	DSM 2782	CpapDRAFT_R0056
Clostridium butyricum	E4 str. BoNT E BL5262	Pathema:CLP_4326
Clostridium sporogenes	ATCC 15579	CLOSPO_00006
Clostridium scindens	ATCC 35704	CLOSCI_03366
Clostridium ljungdahlii	DSM 13528	9443625
Clostridium cellulovorans	743B	9606816

Clostridium saccharolyticum	WM1	Closa_R0002
Clostridium akagii	DSM 12554	AJ237755
Clostridium aldrichii	DSM 6159	X71846
Clostridium caminithermale	DSM 15212	AF458779
Clostridium chartatabidum	DSM 5482	X71850
Clostridium colicanis	DSM 13634	NR_028964
Clostridium drakei	DSM 12750	AJ427628
Clostridium estertheticum	DSM 8809	S46734
Clostridium grantii	DSM 8605	NR_026131
Clostridium hungatei	DSM 14427	AF020429
Clostridium mangenotii	DSM 1289	M59098
Clostridium mayombei	DSM 6539	M62421
Clostridium termitidis	DSM 5398	X71854
Clostridium nitrophenolicum	DSM 21057	AM261414
Saccaharopolyspora erythraea	NRRL 2338	SACE_8101
